# Network Plasticity Involved in the Spread of Neural Activity Within the Rhinal Cortices as Revealed by Voltage-Sensitive Dye Imaging in Mouse Brain Slices

**DOI:** 10.3389/fncel.2019.00020

**Published:** 2019-02-06

**Authors:** Riichi Kajiwara, Yoko Tominaga, Takashi Tominaga

**Affiliations:** ^1^Department of Electronics and Bioinformatics, School of Science and Technology, Meiji University, Kawasaki, Japan; ^2^Laboratory for Neural Circuit Systems, Institute of Neuroscience, Tokushima Bunri University, Sanuki, Japan

**Keywords:** perirhinal cortex, entorhinal cortex, optical imaging, voltage-sensitive dye, plasticity

## Abstract

The rhinal cortices, such as the perirhinal cortex (PC) and the entorhinal cortex (EC), are located within the bidirectional pathway between the neocortex and the hippocampus. Physiological studies indicate that the perirhinal transmission of neocortical inputs to the EC occurs at an extremely low probability, though many anatomical studies indicated strong connections exist in the pathway. Our previous study in rat brain slices indicated that an increase in excitability in deep layers of the PC/EC border initiated the neural activity transfer from the PC to the EC. In the present study, we hypothesized that such changes in network dynamics are not incidental observations but rather due to the plastic features of the perirhinal network, which links with the EC. To confirm this idea, we analyzed the network properties of neural transmission throughout the rhinal cortices and the plastic behavior of the network by performing a single-photon wide-field optical recording technique with a voltage-sensitive dye (VSD) in mouse brain slices of the PC, the EC, and the hippocampus. The low concentration of 4-aminopyridine (4-AP; 40 μM) enhanced neural activity in the PC, which eventually propagated to the EC *via* the deep layers of the PC/EC border. Interestingly, washout of 4-AP was unable to reverse entorhinal activation to the previous state. This change in the network property persisted for more than 1 h. This observation was not limited to the application of 4-AP. Burst stimulation to neurons in the perirhinal deep layers also induced the same change of network property. These results indicate the long-lasting modification of physiological connection between the PC and the EC, suggesting the existence of plasticity in the perirhinal-entorhinal network.

## Introduction

The perirhinal cortex (PC) is a polymodal association area that communicates with neocortical and subcortical areas, such as the sensory, temporal, and insular cortical areas as well as the amygdala, basal ganglia, raphe nucleus, and olfactory bulb (Suzuki, 1996; Kealy and Commins, 2011). Various lesion studies have confirmed that the association between the PC and other brain regions is necessary for fear learning (Corodimas and LeDoux, 1995; Herzog and Otto, 1998; Kholodar-Smith et al., 2008) and object recognition (Murray et al., 1993; Higuchi and Miyashita, 1996; Buckley and Gaffan, 1998). The PC sends axons to the entorhinal cortex (EC) and acts as an important gateway for hippocampal declarative memory processing (Burwell and Amaral, 1998a; Buffalo et al., 1999; Witter et al., 2000; Burwell, 2001; Squire et al., 2004; van Strien et al., 2009; Cappaert et al., 2015). Therefore, the PC operates in conjunction with the hippocampus *via* the EC, especially when spatial or contextual cues are relevant during encoding of object information (Dere et al., 2007). In fact, the one-trial object recognition test, which examines preference behavior for a novel object without reinforcement stimuli, suggests that the PC is especially critical for the encoding of object information and the maintenance of the object memory trace (Winters and Bussey, 2005). In contrast, hippocampal lesions have only revealed impairments when behavioral trials are performed in a complex spatial environment (Winters et al., 2004; Forwood et al., 2005).

The synchronous neural activation *via* the rhinal cortices between the neocortex and the hippocampus is thus suggested to be essential for the formation and recall of context-dependent memories. However, previous electrophysiological experiments in *ex vivo* isolated brains from guinea pigs and *in vivo* anesthetized cats have indicated that propagation of neural activity between the PC and the EC occurs at an extremely low probability (de Curtis and Paré, 2004; Pelletier et al., 2004, 2005). These groups have investigated the physiological and anatomical properties related to PC-EC connectivity (Biella et al., 2001, 2002, 2010; Uva et al., 2005; Pinto et al., 2006; Apergis-Schoute et al., 2007; Unal et al., 2012). Consistent with this phenomenon, using a voltage-sensitive dye (VSD) imaging technique in rat brain slices, we also found that neural activities in response to PC stimulation did not transmit to the EC even under partial suppression of the GABA-A system (Kajiwara et al., 2003). Moreover, we found that associative inputs from the amygdala promoted the spread of perirhinal activity to the EC by activating neurons in the deep layers of the PC/EC, suggesting a functional “gate” that can control information transfer from the PC to the EC. In regard to the mechanism of the gate, further VSD imaging experiments have been conducted by other groups to examine the network between area 35 of the PC and the lateral EC (LEC; Koganezawa et al., 2008; Willems et al., 2016) and have suggested the importance of the inhibitory system in these cortices. More recently, Willems et al. (2018) investigated the role of interneurons in PC/EC transmission by performing whole-cell recordings of interneurons expressing yellow fluorescent protein (YFP) driven by the parvalbumin promotor-dependent cre-recombinase. They revealed that parvalbumin interneurons are involved in eliciting strong inhibition of principal neurons in the deep layer network. These studies focused on the inhibitory system in the PC/EC; however, the contribution of the increase in the excitability of principal neurons is still poorly understood.

In the PC, many excitatory neurons possess late-spiking properties that have been suggested to result from a slowly inactivating potassium conductance (Beggs et al., 2000; Moyer et al., 2002). Since such neurons have delayed and sustained firing properties in response to long synaptic trains, it is difficult for the PC to initiate EC activation. Low concentrations of 4-aminopyridine(4-AP; less than 200 μM) reduce the amplitude of a more slowly inactivating D current while sparing the A current (Rudy, 1988; Storm, 1988; Ficker and Heinemann, 1992; Wu and Barish, 1992; Barish et al., 1996). In this article, we examined the effect of a low concentration of 4-AP on the gate, which normally blocks neural transmission from the PC to the EC.

In a preliminary experiment on PC-EC transmission using the VSD imaging method, we noticed that once the PC/EC gate was open, even weak stimulation of the PC easily activated the EC network, and this property persisted throughout the experiment. Recent electrophysiological and VSD imaging studies in isolated whole brains from guinea pigs revealed that paired theta frequency stimulation of two distant neocortical sites resulted in a prolonged response potentiation to the paired inputs (Unal et al., 2012). However, persistent modification of the physiological connectivity between the PC and the EC was not mentioned because the potentiated response in the PC did not drive the entorhinal activity. Here, we also aimed to determine whether plastic changes in PC-EC transmission occur.

A single-photon wide-field optical recording technique can be used to analyze such plastic changes in network dynamics by elucidating what types of changes occur and identifying the critical region for these changes. However, to observe long-lasting modification of the network property, stability to fix the inherent problems of VSD imaging (e.g., toxicity and bleaching of the dye) is required. In the present study, by using a low-magnification epi-fluorescent macroscope to maximize optical efficacy (Tominaga et al., 2018), the long-term transition of the state in the network activity in a slice was stably visualized, and we investigated the plastic behavior in the PC-EC network.

## Materials and Methods

All procedures involving animal experiments were approved by the Animal Care and Use Committee of Tokushima Bunri University and Meiji University.

### Brain Slice Preparation and VSD Staining

Adult male C57BL/6 mice aged 12–36 weeks were decapitated under deep isoflurane anesthesia. The brains were quickly cooled in ice-cold oxygenated artificial cerebrospinal fluid (ACSF) containing the following in mM: 124 NaCl, 2.5 KCl, 2 CaCl_2_, 2 MgSO_4_, 1.25 NaH_2_PO_4_, 26 NaHCO_3_, and 10 glucose with a pH of 7.4 when saturated with a mixture of 95% O_2_ and 5% CO_2_. After cooling for 5 min, the cerebellum, the brainstem and the frontal third of the brain were removed, and horizontal slices (350 μm) containing the PC, the EC [including the medial EC (MEC) and the LEC], and the hippocampus were prepared using a vibrating blade microtome (VT1200S, Leica) as described previously (Kajiwara et al., 2003). Each slice was transferred onto a fine-mesh membrane filter (Omni Pore membrane filter, JHWP01300, Millipore) held in place by a thin plexiglass ring (inner diameter, 11 mm; outer diameter, 15 mm, thickness 1–2 mm; Tominaga et al., 2000). Slices placed in the plexiglass ring were transferred to a moist holding chamber continuously supplied with ACSF infused with the O_2_ and CO_2_ gas mixture. After 1 h of incubation in this chamber, slices were bath-stained for 25 min with an aliquot of VSD staining solution (100 μl for each slice), consisting of 0.2 mM aminonaphthylethenyl-pyridinium (Di-4-ANEPPS; D-1199, Invitrogen) in 2.7% ethanol, 0.13% Cremophor EL (Sigma-Aldrich Co.), 50% fetal bovine serum (Sigma-Aldrich Co.), and 50% ACSF. The slices were subjected to experiments after incubation at room temperature in normal ACSF for at least 1 h.

### Optical Recording System and Data Processing

The plexiglass ring supporting each slice was placed in an immersion-type recording chamber. Slices were continuously perfused with prewarmed (31°C) and oxygenated ACSF (bubbled with a 95%/5% O_2_/CO_2_ gas mixture) at a rate of 1 ml/min. Custom laboratory-designed epifluorescence optics consisting of two principal lenses were used to view the slices during experiments. The optics consisted of an objective lens for the microscope (×1.6 Leica Micro Systems MZ-APO) and another lens (×0.63 Leica Microsystems MZ-APO) as the projection lens. Excitation light was provided by a halogen lamp source (150 W; MHW-G150LR; Moritex Corp.) projected through an excitation filter (*λ* = 530 ± 10 nm) and reflected onto the hippocampal slice by a dichroic mirror (*λ* = 575 nm). Emission fluorescence from the slice was passed through an emission filter (*λ* > 590 nm) and projected onto a cMOS camera (MiCAM Ultima; BrainVision, Inc., Tokyo, Japan). Most recordings were made at a frame rate of 0.5 ms/frame.

The fluorescence intensity emitted by the slice prior to stimulation (a prestimulation period usually lasting from 100 to 740 frames) was averaged and used as the reference intensity (F0). The fractional change in fluorescence [ΔF(t) = F(t) − F0] was normalized by the F0 (ΔF/F0) and used as the optical signal. The optical signals referred to in the following sections represent signals filtered in spatial and temporal dimensions with a digital Gaussian kernel of 5 × 5 × 3 (horizontal × vertical × temporal; σ ≈ 1). In some experiments, we observed a slow drift in the baseline signal; in these cases, the drift was compensated for by subtracting a normalized smooth spline curve obtained from optical signals recorded at pixels where no response was observed. We confirmed that this procedure produced steady and flat baseline signals and did not cause an artificial drift in the absence of stimulation.

Recordings were made in a total of 45 slices, and we analyzed optical signals offline using a procedure developed for Igor Pro (WaveMetrics Inc., Portland, OR, USA). The data presented here are drawn from a pool of 29 successful experiments. At a wavelength of 610 nm, the VSD fluorescence decreases in response to depolarization of the membrane. To fit the polarity of the response to conventional membrane potential changes, we expressed the optical signal in a polarity that matched the membrane potential change. For example, decreased fluorescence, which corresponds to depolarization, was represented as a positive deflection. In some experiments, regions of interest (ROIs) consisting of 21–23 VSD signals were selected. The average of the maximum VSD signal amplitudes and the standard deviation of each ROI were calculated. For additional details on the methods, see Tominaga et al. (2000, 2002).

### Stimulation and Field Potential Recording

To investigate the influence of the network activity in the PC on the behavior of EC neurons, we stimulated various sites using a stimulus intensity that caused the maximum response of superficial layers in the PC (100–150 μA for 200 μs, bi-phasic pulse). The position of the stimulating electrode was changed from the rostral (area 36 of the PC) to the caudal area (area 35) close to the boundary between the PC and the EC. The stimulating electrodes were glass electrodes filled with ACSF (1 MΩ). In some experiments, field potential recordings were performed to confirm no photodynamic effect on the field potential amplitude during the optical recording. A glass recording electrode filled with ACSF (1 MΩ, 5-μm inner diameter) was positioned in the entorhinal and perirhinal layers II/III near the rhinal sulcus. A laboratory-made differential amplifier and a general-purpose amplifier (model 440; Brownlee Precision, San Jose, CA, USA) were used for field potential recordings (100× gain, bandpass DC–3 kHz).

### Drugs

A large number of parvalbumin immunoreactive neurons have been observed on the border of the perirhinal area and in the EC (Wouterlood et al., 1995; Miettinen et al., 1996). To investigate the spatiotemporal distribution of excitatory activities between the PC and the EC, synaptic inhibition was partly suppressed by applying a low concentration of the GABA-A antagonist gabazine (0.5–1 μM; SR95531, Sigma-Aldrich Co.). Under such partial suppression of the inhibitory system, we observed perirhinal–entorhinal neural activation without inducing spontaneous paroxysmal activities in rat brain slices (Kajiwara et al., 2003).

In the PC, there are many excitatory neurons possessing late-spiking properties suggested to result from a slowly inactivating potassium conductance (Beggs et al., 2000; Moyer et al., 2002). In some experiments, we perfused brain slices with 40 μM 4-AP (Sigma-Aldrich Co.) to increase the firing rate of principal neurons by blocking potassium channels. 4-AP is a potassium channel blocker that affects A-type (Gutman et al., 2005), D-type (Storm, 1988) and delayed rectifier potassium channels (Gutman et al., 2005). Low concentrations of 4-AP (less than 200 μM) reduce the amplitude of a more slowly inactivating D current while sparing the A current (Rudy, 1988; Storm, 1988; Ficker and Heinemann, 1992; Wu and Barish, 1992; Barish et al., 1996).

## Results

### The Presence of 0.5 μM Gabazine in the Recording Solution Allowed us to Examine the Behavior of the Gate

**Figure 1:**
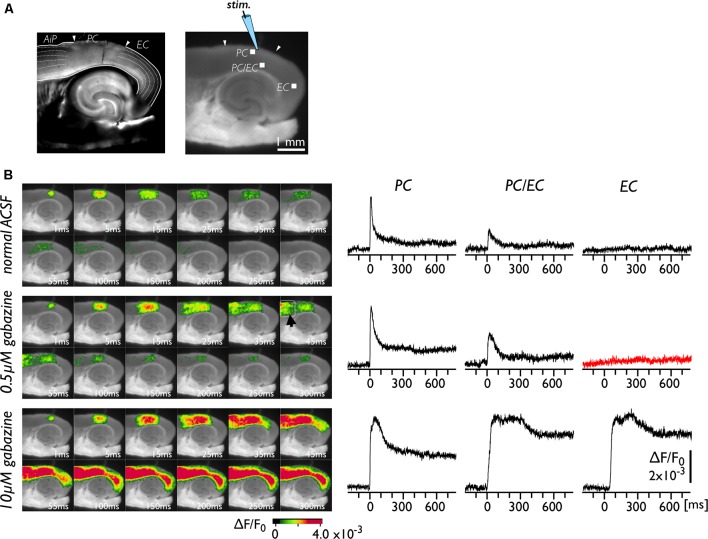
Spread of neural activity upon single stimulation of perirhinal cortex (PC) under gabazine treatment. **(A)** Bright-field (left) and fluorescent (right) images of mouse horizontal brain slices. This type of brain slice includes the hippocampus, the entorhinal cortex (EC), the PC, and the insular cortex (AiP). The stimulus electrode was placed in superficial layers in the PC, and the membrane depolarization change was recorded. **(B)** Typical spread pattern of evoked neural activity following electrical stimulation. Sequence of images are the neural activity map after the stimulation obtained under normal solution (upper), 0.5 μM (middle) and 10 μM gabazine treatment (bottom). Depolarization was measured as fractional changes in fluorescence in each pixel; this value is encoded in pseudocolor as indicated in the scale and superimposed on a fluorescent image of the slice. Voltage-sensitive dye (VSD) signal traces of the PC, the PC/EC and the EC in response to the single stimulation are also presented. Electrical stimulation elicited a prolonged response in the EC under 10 μM gabazine (bottom) but did not evoke the EC response under partial suppression of the GABA-A system (0.5 μM; red-trace).

The neural transmission from the PC to the EC was tested under the perfusion of gabazine, which blocks GABA-A-dependent inhibitory transmission in the entire network of the brain slice. Figure 1A represents bright-field (left) and fluorescent (right) images of brain slices. The positions of the PC, the EC and the insular cortex (AiP) were identified from the left image with reference to previous literature (Kirkcaldie, 2012; Sills et al., 2012; Willems et al., 2016). A representative experiment is illustrated in Figure 1B. We recorded the change in VSD signals in slices following stimulation of the superficial layers of the PC (Figure 1A). Under normal conditions (Figure 1B, upper), entorhinal activity was not activated by PC stimulation. Because the propagation of neural activity in a slice can be suppressed by local inhibitory neurotransmission (Iijima et al., 1996), we perfused slices with 0.5 μM gabazine (Figure 1B, middle). As a result, the evoked neural activity propagated to the AiP (arrowhead in the 45 ms image) but not to the EC (red trace). However, by increasing the concentration of gabazine to 10 μM, we observed apparent neural activity transmission between the PC and the EC (Figure 1B, bottom). We performed similar experiments (*n* = 25) and confirmed that 0.5 μM gabazine did not cause entry of perirhinal activity into the EC in our experimental conditions. Hereafter, we used 0.5 μM gabazine to investigate the gate between the PC and the EC.

### Effect of Low-Concentration 4-AP on Neuronal Propagation Across the PC/EC Border

**Figure 2:**
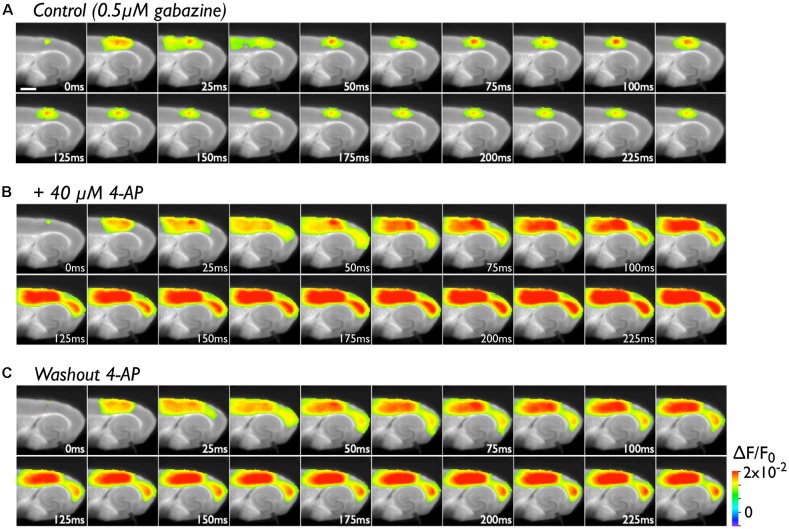
The effect of 4-aminopyridine (4-AP) treatment (40 μM) on the initiation of excitation propagation from the PC to the EC. **(A)** Consecutive images showing the spread of neural activity through the cortices upon burst stimulation (40 Hz, 5 pulses) to superficial layers of the PC under the perfusion of gabazine (0.5 μM). The numbers on each image indicate the time after stimulation in milliseconds in the presence of 0.5 μM gabazine. **(B)** The propagation pattern under the coperfusion of 4-AP (40 μM) and gabazine to the same stimulation. **(C)** After 4-AP washout.

**Figure 3:**
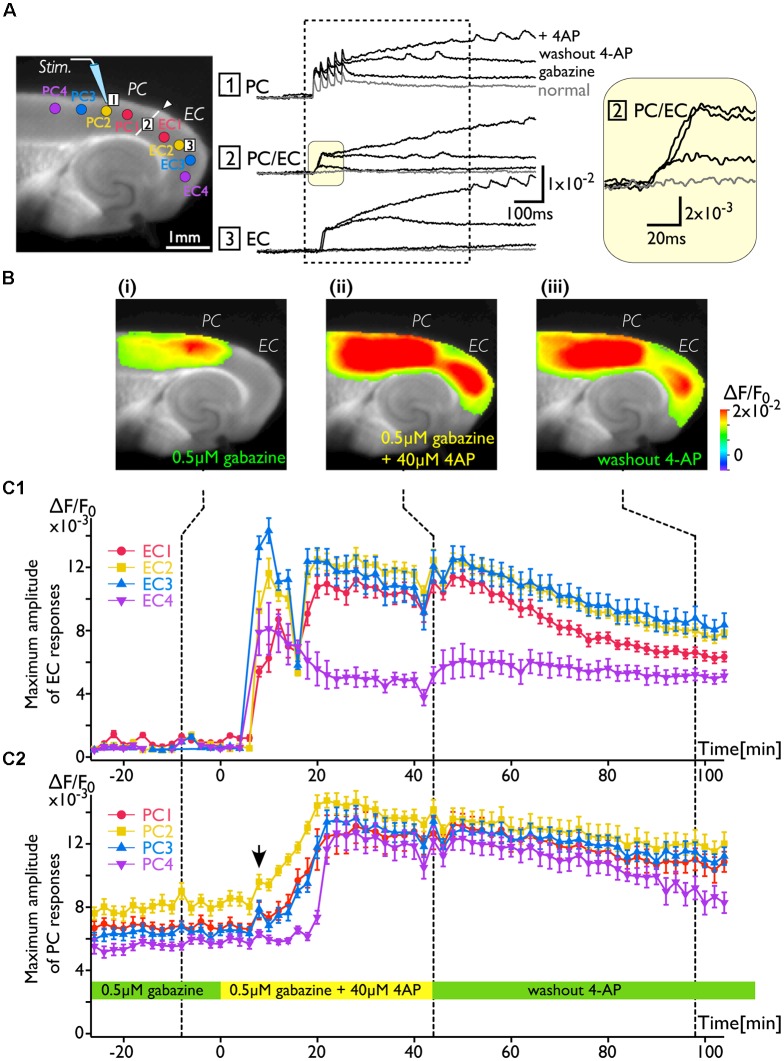
The change in the PC-EC network property caused by 4-AP application. VSD imaging was performed every 2 min for 2 h. The recording solution contained gabazine (0.5 μM) throughout the experiment. Perfusion of 4-AP (40 μM) started at 0 ms, and washout started at 44 min. **(A)** The position of the stimulating electrode in the PC is shown in the fluorescent image. Traces are the VSD signals recorded from: (1) the PC; (2) the PC/EC border; and (3) the EC. The VSD signals recorded at −8, 44, and 98 min were superimposed. The inset on the right indicates the initial phase of the depolarization in the PC/EC on an expanded scale. Regions of interest (ROIs) consisting of 21–23 VSD signals were selected from PC1, 2, 3, and 4 and EC1, 2, 3, and 4 (colored circle in the image of **A**). The average of maximum VSD signal amplitudes of 21–23 pixels at each ROI were calculated during the period indicated by the dotted rectangle and plotted in **(C)**. **(B)** Representative maximum response map obtained from the acquisition at the time indicated by the dotted line (**i**: −8 min, **ii**: 44 min, and **iii**: 98 min) in **(C)**. **(C)** Average maximum amplitude plots of ROIs in the EC **(C1)** and the PC **(C2)** throughout the experiment. The error bar represents the standard deviation of maximum amplitudes in a ROI. The averaged maximum value of each ROI displayed a significant increase before and after 4-AP perfusion (*P* values determined using the *t*-test ≪10^−3^).

The PC contains many late-spiking neurons, which characteristically possess slowly inactivating potassium channels (Faulkner and Brown, 1999; Beggs et al., 2000; McGann et al., 2001; Moyer et al., 2002). Such neurons might contribute to the blockade of neural transmission to the EC in conjunction with the PC/EC inhibitory system. To obtain evidence in support of this idea, we perfused slices with 40 μM 4-AP in the presence of 0.5 μM gabazine. In the control experiment, we stimulated (five pulses at 40 Hz) the superficial layers of the PC near the PC/EC border (Figure 2A). The responses elicited by a stimulating electrode mainly propagate in the rostrocaudal direction within the stimulated superficial layers for 25 ms. During this period, activity in the superficial layers also propagated to the deep layers of the PC. Repetitive inputs did not cause neural propagation across the PC/EC border. However, entorhinal activity was observed after co-application of 4-AP with gabazine (Figure 2B). In this set of experiments, five pulses at 40 Hz were applied to the superficial layers of the PC. Such repetitive pulses caused an increase in the amplitude of perirhinal responses (the VSD signal traces shown in Figure 3A) in the presence of 4-AP and gabazine that persisted even after cessation of the 40-Hz stimulus. Moreover, enhancement of the neural activity in the PC triggered neural propagation to the EC. Even after washout of 4-AP, excitation propagation from the PC to the EC was still observed (Figure 2C), and it lasted for more than 50 min. Thus, the boundary area between the PC and the EC seems to act as a “gate” for information transfer from the PC to the entorhinal-hippocampal circuit. The data presented here are representative of nine similar experiments.

### Long-Term Continuous VSD Imaging of the Neural Activity Propagation Caused by 4-AP

As shown in Figure 2, the increase in excitability at the PC/EC border region induced by 4-AP application caused perirhinal-entorhinal transmission, although partial blockade of GABA-A receptors did not. Our hypothesis is that once the PC/EC gate is open, the PC response can propagate to the EC even when 4-AP is washed out. To investigate the transition of PC/EC network property throughout the experiment, the optical recording system must be sufficiently stable (Barish et al., 1996). By improving the system in many aspects (e.g., optics, staining protocol and performance of the camera), we were able to maintain the slice in a stable condition (Barish et al., 1996; Tominaga et al., 2013; Tominaga and Tominaga, 2016). Thus, we continuously acquired a sequence of images every 2 min for 2 h. Figure 3 indicates that the 4-AP-induced change in the PC-EC propagation pattern persisted following 4-AP washout. Each image shown in Figure 3B is the maximum response map calculated from a sequence of images and shows that the EC was activated after perfusion of 4-AP. Furthermore, we averaged the maximum amplitude of 21–23 pixels at some ROIs selected from the PC and the EC (Figure 3A) as an index of the degree of activation in the PC and the EC, respectively. Figures 3C1,2 illustrate the time series graph in which the maximum values of each ROI were plotted. The averaged maximum value of each ROI displayed a significant increase between before and after 4-AP perfusion (*P* values determined using *t*-test ≪ 10^−3^). During the experiment, 4-AP was added to the recording solution from 0 to 40 min and then washed out. The maximum response plot of the EC in Figure 3C1 indicates that 4-AP led to a rapid rise and fall in the EC response (blue and yellow plots) between 8 and 16 min after the initiation of 4-AP perfusion (0 min). During this period, small transient changes (allow head) following the gradual increase were observed in the PC (Figure 3C2). From 44 to 100 min, 4-AP was washed out of the solution; however, EC activation was still observed (Figure 3Biii), although activation of both the EC and the PC was gradually reduced. At the most medial part of the EC, the value remained stable (purple plots in Figure 3C1).

### Neural Propagation From the PC to the EC Caused by Repeated Deep Layer Stimulation at 40 Hz

**Figure 4:**
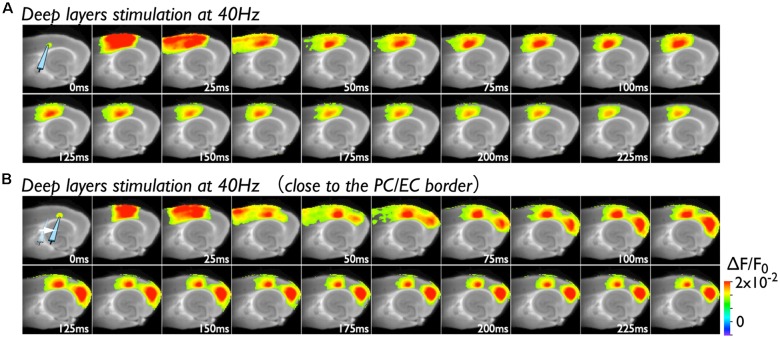
Spread of the neural activity from the PC to the EC elicited by deep layer stimulation in the presence of 0.5 μM gabazine. **(A)** The time sequence of the burst stimulation (40 Hz, 5 pulses) to deep layers. The position of the stimulating electrode in the PC is shown in the image at 0 ms. **(B)** Spread of neuronal activity caused by stimulation of the deep layers. The position of the stimulation electrode was moved to the adjacent area of the PC/EC border.

**Figure 5:**
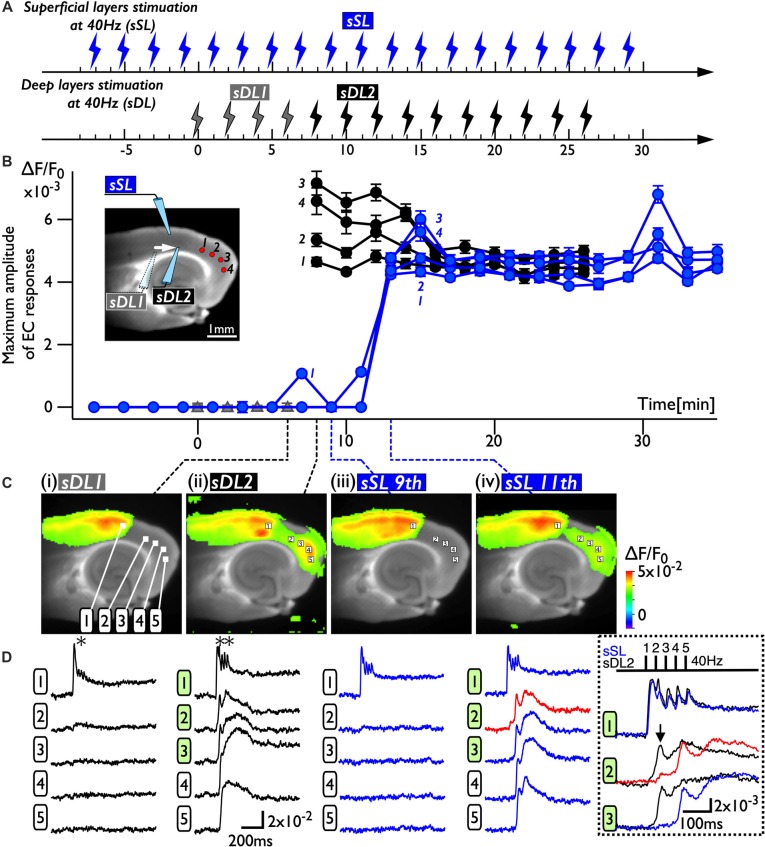
The change in the PC-EC network property caused by alternating stimulation of deep and superficial layers. **(A)** The stimulation protocol of the experiment. Burst stimulation (5 pulses at 40 Hz) was applied to the superficial layers (sSL) and deep layers (sDL1 and sDL2) in the PC. At 0, 2, 4, and 6 min, sDL1 was applied, and sDL2 was applied after 8 min. sSL and sDL were alternately applied every 1 min. **(B)** Maximum amplitude plots of ROIs in the EC and a fluorescent image of the slice. The position of the stimulating electrode is indicated in the fluorescent image. The positions of the ROIs are indicated by red circles. The temporal variability of the maximum response induced by stimulation of the deep (black) and superficial (blue) layers is plotted in black and blue, respectively. **(C,D)** Maximum response map **(C)** and representative temporal pattern of the response **(D)** induced by the stimulation of deep layers (**i**: sDL1, **ii**: sDL2) and superficial layers (**iii** and **iv**: sSL9th and sSL 11th). Each number on the image indicates the pixel of the VSD signals extracted. The traces shown in the dotted rectangle indicate the initial phase of the responses in **(ii)** and **(iv)** on an expanded time scale. The five pulses at 40 Hz were applied as indicated above. Black and blue traces represent the response to stimulation of deep layers and superficial layers, respectively. The response at the PC/EC border in response to stimulation of superficial layers is indicated by red.

Because deep layers of the perirhinal area 35 neighboring the EC have been reported to play a key role in neural propagation from the PC to the EC (Kajiwara et al., 2003), we examined the impact of deep layer excitability on the gating property by applying 40 Hz stimulation (Figure 4). Initially, we were not able to record neural excitation propagation across the PC/EC border (Figure 4A). As shown in Figure 4B, however, the increase in excitability at the PC/EC border region caused perirhinal-entorhinal transmission when the position of the stimulation electrode was moved to the PC/EC border (Figure 4B). We postulated that once the excitability of neurons at the PC/EC border increased to the level needed to initiate PC-EC transmission, inputs to the superficial layers also transmitted to the EC. We confirmed this hypothesis by the continuous acquisition of the neural activity evoked by alternately applied stimulation to deep or superficial layers every 1 min (Figure 5). In this experiment, we placed the stimulating electrodes in both the superficial (sSL) and deep layers (sDL1 or sDL2) of the PC and investigated the contribution of each stimulus to the change in the network response pattern over time according to the stimulation protocol indicated in Figure 5A. Regarding the sDL, the position of the electrode was moved from sDL1 to sDL2 (close to the border) at 8 min.

Figure 5B represents a maximum amplitude plot of the EC response at ROIs shown in the inset image. EC responses were clearly elicited by sDL2, that is, PC-EC transmission occurred. In addition, sSL elicited an entorhinal response after 13 min, which persisted throughout the experiment. Figures 5C,D illustrate the maximum response map and examples of optical signal traces extracted from the PC (trace 1), the PC/EC border (trace 2), and the EC (trace 3, 4, 5). A five-pulse 40-Hz stimulation by sDL1 elicited a sharp large-amplitude response (asterisk) in the PC, whereas a noticeable response in the EC was not observed. However, sDL2 elicited a transient sharp response followed by a slow large-amplitude response at the PC/EC border (trace 2) and in the EC (trace 3, 4), showing an effect of repetitive stimulation (double-asterisk). As shown in Figure 5Civ, an EC response was elicited by subsequent application of stimulation to the superficial layers (11th sSL), although not immediately (9th sSL). The optical signal traces of Figures 5Cii,iv on an expanded time scale shown in the dotted rectangle in Figure 5D indicate that the 1st and/or 2nd pulse of repetitive stimulation of sDL1 contributed to initiation of the EC response (arrow on the black-trace). In contrast, repetitive (1st, 2nd, and 3rd) inputs by the sSL increased the excitability of neurons at the PC/EC border (red-trace 2) and evoked the large-amplitude response in the EC (blue-trace 3) as well as at the PC/EC border itself.

## Discussion

This study addressed the long-term modification of network activity, termed network plasticity, in mouse perirhinal/entorhinal cortical slices using a recently developed VSD imaging technique (Tominaga et al., 2018). By implementing a low-magnification lens with a high-N.A. in the system, we analyzed the neural activity in a wider field of view and minimized the impact of bleaching of the dye on the long-term recording. The stability of the experimental system enabled us to investigate the dynamics of neural transmission *via* the “gate” in the PC/EC over a long time period and contributed to new findings. First, the increase in neuronal excitability caused by bath application of 4-AP initiated the transmission of perirhinal activity to the EC under conditions in which GABA-A control partially remained. Second, once neural propagation across the perirhinal/entorhinal border was elicited, the network property was changed, and perirhinal neural activity easily propagated to the EC even after 4-AP washout. Third, such a change in the network property was accomplished without 4-AP treatment using repeated burst stimulation of neurons in the deep layers of the PC to initiate neural propagation across the border. Consequently, even ineffective stimulation of the superficial layers of the PC was effective for neural transmission to the EC.

### Inhibitory Control of Neural Propagation Across the Rhinal Sulcus

Based on observations obtained from electrophysiological and imaging studies performed on various brain preparations, such as *in vivo* (Pelletier et al., 2004) and *ex vivo* isolated whole brain (Biella et al., 2002) and brain slice (Kajiwara et al., 2003) preparations, the local network between the PC and the EC is considered to form a wall of inhibition that blocks reciprocal information transfer (de Curtis and Paré, 2004). One of the factors that causes impaired neuronal transmission from the PC to the EC despite the existence of anatomical connections within the PC/EC network is the inhibitory system. Pelletier et al. (2005) reported that repetitive neocortical stimuli or associative stimulus by a conditioning shock to the amygdala enhanced the amplitude of neocortically evoked field potentials in the PC; however, this increased excitability did not augment the responsiveness of entorhinal neurons. They suggested that the increased excitatory drive in the PC was insufficient to overcome the local inhibition. Consistent with this, propagation of perirhinal activity to the EC was observed with a high concentration (10 μM) of a GABA-A antagonist (Figure 1B, bottom). However, when the concentration of gabazine was 0.5 μM, entorhinal activity was not evoked by perirhinal stimulation, although neural propagation to the AiP was observed (Figure 1B, middle). These observations imply the existence of a unique network structure consisting of different types of inhibitory systems in the PC-EC region. Evidently, a large number of parvalbumin immunoreactive neurons are located in the EC, especially near the border to the PC (Wouterlood et al., 1995; Miettinen et al., 1996). Meanwhile, the PC possesses specific interneuronal structures, with an unusually high proportion of calretinin-positive interneurons (Barinka et al., 2012). Moreover, a combined study of anterograde tracing and GABA immunocytochemistry techniques revealed the characteristic inhibitory networks that should control the impulse traffic from the PC to the EC (Pinto et al., 2006; Apergis-Schoute et al., 2007). Such an inhibitory system at the boundary area between the PC and the EC may be critical for the gate, affecting the information transfer from the PC to the entorhinal-hippocampal circuit (Maurer et al., 2017).

### 4-AP Can Open the Gate for the Neural Transmission From the PC to the EC

The pathway between the PC and the EC is thus under control of the inhibitory system and might form a gate for information transfer from the neocortex to the hippocampus. In addition to disinhibition of the inhibitory system, hyperexcitability of principal neurons in the PC may also affect gate opening. Indeed, even under the weak inhibitory control condition achieved by partial blockade of the GABA-A system, we observed PC-EC transmission by applying associative stimulation, which caused an increase in the excitability of PC neurons (Kajiwara et al., 2003). Because there are many late-spiking neurons in the PC (Faulkner and Brown, 1999; Beggs et al., 2000; McGann et al., 2001; Moyer et al., 2002), a low concentration of 4-AP (40 μM) may have affected the slowly inactivating potassium conductance of these neurons, inducing rapid depolarization of the membrane potential. Certainly, we confirmed that 4-AP application in a solution containing 0.5 μM gabazine induced perirhinal-entorhinal neural transmission (Figure 2B). Similar PC-EC propagation has been reported in the ictal/interictal epileptic conditions induced by 4-AP, bicuculine and/or low Mg^2+^ concentrations (Stoop and Pralong, 2000; de Guzman et al., 2004; Zahn et al., 2008). However, it was difficult to identify the critical region for propagation *via* the PC/EC gate because of the limitation of the spatial resolution in electrophysiological methods. By performing the VSD imaging technique in the present study, we successfully observed an increase in the depolarized response in deep layers of the PC/EC (*see the image at 25 ms in Figure 2B*), suggesting that the physiological connectivity in this area was increased.

### An Increase in the Excitability of Deep Layers at the PC/EC Border Is Necessary for the Gate to Open

Under 4-AP perfusion, the neural activity in response to superficial layer stimulation in the PC propagated to the EC *via* the deep layers at the PC/EC gate. Deep layer stimulation without 4-AP evoked the propagation of the neural response to the EC (Figure 2Bii). These results correspond to our previous study performed in rat brain slices (Kajiwara et al., 2003), indicating that repetitive stimulation at 40 Hz of deep layers near the PC/EC border (area 35) triggered the EC response, but stimulation of superficial layers did not. Anatomical studies have indicated that the PC projects heavily to the EC, especially the lateral part (Cappaert et al., 2015), and the projection from the PC terminates preferentially in the superficial layers of the EC (Naber et al., 1997; Burwell and Amaral, 1998b). Why was prominent excitation first observed in deep layers in our studies? A likely explanation is that the VSD imaging in the present study revealed a physiologically activated multi-synaptic pathway, whereas typical anterograde/retrograde tracing studies reveal the majority of the projection fibers, dendrites and cell bodies of principal neurons. Layers II and III of the EC contain a dramatically higher density of parvalbumin-positive fibers, dendrites, and cell bodies than those of the PC (Wouterlood et al., 1995; Miettinen et al., 1996). In our experimental conditions, the remaining GABA-A-mediated transmission by these inhibitory neurons might affect PC-EC neural propagation. Moreover, the PC-EC network is thought to be controlled by a strong feed-forward inhibitory system (Pinto et al., 2006), including various modes, e.g.,: (1) principal cells in perirhinal area 36 (far from the border of the PC/EC) and area 35 (near the border) projecting to inhibitory neurons in each area sequentially (stepwise inhibition); (2) neurons projecting beyond the adjoining area (leap mode); and (3) axons of GABAergic cells extending beyond the area where their soma is located (long-range inhibition). Because the superficial EC neurons receive such multisynaptic innervations as well as direct projections from the PC, deep layers at the PC/EC border under relatively weak inhibitory control may be an alternative pathway to the EC.

### Plasticity of the Neuronal Network in the Rhinal Cortices

As we described above, repetitive 40-Hz stimulation to the deep layers of the PC triggered neural transmission across the PC/EC gate (Figure 4B), potentially due to a change in the multisynaptic network involving the transfer of neural activity to the EC from a *steady state* (degree of activation in the PC and EC is “high” and “low”) to an *active state* (“high” and “high”;* i.e., gate-open)*. In support of this idea, superficial layer stimulation did not cause neural transmission across the PC/EC border in the steady state (Figures 2B, 5Ciii). However, once the state was changed to active by deep layer stimulation (Figure 5Cii), stimulation of the superficial layers with the same intensity was able to cause an entorhinal response (Figure 5Civ). In the active state, repetitive stimulation of the superficial layers was able to depolarize the membrane potential of neurons in the PC/EC border (Figure 5D, red-trace). The delay between the onset and the first peak of the optical response elicited by the stimuli to the superficial layers was approximately 100 ms, which is longer than that elicited by deep layer stimulation (30 ms). During this delay, the membrane slowly depolarized. This slow depolarization may be caused by late-spiking neurons in the PC and may be shortened by 4-AP application. Thus, 4-AP was also able to change the state to active and trigger PC-EC neural propagation (Figures 2, 3). In our experimental condition, such a change in the state persisted for a long time, as indicated in Figures 3C, 5B, suggesting the existence of network-scale plasticity in the rhinal cortices, especially at the PC/EC border.

One of the most fundamental principles of plasticity is Hebb’s law. In his book, Hebb (1949) suggests that the two adjacent areas will begin to activate together once a plastic change occurs. Our finding that the repetitive stimulation of the deep layer of the PC causes a persistent change in circuit behavior that allows the spread of activity across the PC/EC border is similar to Hebb’s assumption. Hence, we can also assume that multiple excitatory and inhibitory neurons in this area, probably within the deep layer of the PC/EC border, would exhibit plastic connections. We cannot determine the precise mechanism of plasticity. However, application of a small amount of 4-AP induced the same plastic change, suggesting the involvement of late-spiking neurons in this area. de Guzman et al. (2004) showed 4-AP-induced ictal-like spontaneous activity, which was completely blocked by the addition of NMDA antagonist in the PC, by performing field potential recording of brain slices in an interface-type chamber. In our experimental condition, we did not observe such activity, however, under the current protocol (i.e., perirhinal stimulation under perfusion of 40 μM 4-AP and 0.5 μM gabazine), we infrequently observed the evoked oscillatory activity in the restricted region of the PC/EC border at a frequency range from 7 to 40 Hz (unpublished data). In addition, the PC is suggested to be implicated in the processes of epileptogenesis and ictogenesis (Biagini et al., 2013). Thus, the local circuit in the PC, which possesses the ability to induce oscillating activity, may affect the network plasticity through the rhinal cortices. In fact, Zarnadze et al. (2016) reported that gamma rhythms lead to activity-dependent modification of hippocampal networks, and this plasticity was metabotropic glutamate receptor 5 dependent. The plasticity might also involve plastic changes in gap junctions, which have been suggested to exist in the PC (Zlomuzica et al., 2010) and may synchronize neuronal activity and/or accelerate neuronal transmission. Moreover, because synchronous activation of astrocytes has been observed in the rhinal cortices during the spread of epileptic activities (Losi et al., 2016), astrocytes as well as neurons may also be involved in the observed phenomena. Although further experiments are required to confirm this speculative idea, the stable VSD imaging technique with a wide field of view revealed that network-level plastic changes occurred in the PC/EC region, thereby generating new insights into plasticity in this region.

## Author Contributions

RK and TT designed the research and wrote the article. TT, YT, and RK performed the research and analyzed the data. YT and TT developed the software.

## Conflict of Interest Statement

The authors declare that the research was conducted in the absence of any commercial or financial relationships that could be construed as a potential conflict of interest.
